# WiFi Indoor Localization with CSI Fingerprinting-Based Random Forest

**DOI:** 10.3390/s18092869

**Published:** 2018-08-31

**Authors:** Yanzhao Wang, Chundi Xiu, Xuanli Zhang, Dongkai Yang

**Affiliations:** School of Electronic and Information Engineering, Beihang University, Beijing 100191, China; xuanli_zhang@foxmail.com (X.Z.); yangdongkai@sina.com (D.Y.)

**Keywords:** channel state information (CSI), Random Forest, fingerprinting, indoor positioning, WiFi

## Abstract

WiFi fingerprinting indoor positioning systems have extensive applied prospects. However, a vast amount of data in a particular environment has to be gathered to establish a fingerprinting database. Deficiencies of these systems are the lack of universality of multipath effects and a burden of heavy workload on fingerprint storage. Thus, this paper presents a novel Random Forest fingerprinting localization (RFFP) method using channel state information (CSI), which utilizes the Random Forest model trained in the offline stage as fingerprints in order to economize memory space and possess a good anti-multipath characteristic. Furthermore, a series of specific experiments are conducted in a microwave anechoic chamber and an office to detail the localization performance of RFFP with different wireless channel circumstances, system parameters, algorithms, and input datasets. In addition, compared with other algorithms including K-Nearest-Neighbor (KNN), Weighted K-Nearest-Neighbor (WKNN), REPTree, CART, and J48, the RFFP method provides far greater classification accuracy as well as lower mean location error. The proposed method offers outstanding comprehensive performance including accuracy, robustness, low workload, and better anti-multipath-fading.

## 1. Introduction

With the increasing proliferation rate of smart devices, Location Based Services (LBSs) have gained considerable attention. The Global Positioning System (GPS), which has been widely applied in outdoor localization, has profited enormously from the line of sight (LOS) transmission channels between satellites and the receiver in the outdoor world [1], but the localization accuracy will sharply decline once the receiver enters a non-line of sight (NLOS) environment. Therefore, Indoor Positioning Systems (IPSs) have been extensively investigated and intense efforts have been devoted to enhance the localization performance [2,3,4,5].

WiFi indoor localization has been studied largely in recent years. Considering that the utilization of current existing IEEE 802.11 network infrastructures is widespread in massive numbers of residential areas, office spaces, and commercial districts, the fingerprinting-based localization which utilizes Receive Signal Strength (RSS) data has become one of the most popular indoor positioning systems. The fingerprinting-based localization is composed by two basic stages: one is an offline training stage and the other is an online testing stage. During the offline training stage, RSS data are collected at each receiver point (RP) by an acquisition procedure from all detectable access points (APs). Unlike other localization systems, for instance, Radio Frequency Identification (RFID) [6,7], Bluetooth [8,9], Ultra-Wideband (UWB) [10,11] and Simultaneous Localization and Mapping (SLAM) [12], which strongly depend on extra devices, WiFi indoor localization utilizes widespread WiFi routes as APs and brings enormous convenience to database construction. Diverse machine learning methods are employed after RSS data collection to obtain outstanding localization performances, including high accuracy, low complexity, and short matching times. In the online testing stage, the real-time RSS data recorded by the user’s mobile device is used to estimate his/her location and the location of the tester by matching with the offline fingerprint database. Single classifiers, such as K-Nearest-Neighbors (KNN), and machine learning methods, such as Back Propagation Neural Networks (BPNN) and Support Vector Machine (SVM), have been applied for fingerprinting-based indoor localization. The KNN algorithm chooses *k* fingerprints with the minimum Euclidean distances to the instantly reported RSS vector among different fingerprints in the locally stored radio map. The first *k* RPs resulting in the minimum Euclidean distances are used to derive the estimated location [13]. One downside to this algorithm is that it needs to store all of the RSS training values, and another disadvantage of KNN is that the localization accuracy is largely dependent on the value of *k*. BPNN has been employed into the positioning applications. Through a Back Propagation training process, the well-trained neural model then can be used to estimate the object’s location based on the RSS measurements. SVM, on the other hand, employs Kernel functions to improve the randomness and incompleteness of the RSS values, but with the cost of high computational complexity [14,15]. The ensemble of classifiers, also known as the ensemble algorithm, combines various simple classifiers into a unified and integrated classifier with better stability and accuracy performance than any individual learner. Hansen and Salamon proposed a set of neural networks with the ensemble consensus mechanism [16]. Then, Breiman proposed the Random Forest method [17]. In the field of indoor positioning, Reference [18] presents a generic AdaBoost framework with a robust threshold mechanism and structural optimization on regression problems and analyzes the bounds of the generalization error directly under probably approximately correct learning. A real-world indoor positioning application revealed that the proposed method has higher positioning accuracy and faster speed. Reference [19] proposes a strategy which incorporates a preprocessing method for RSS samples, the implicit crowdsourcing sampling technique, and a semi-supervised learning algorithm. Reference [20] introduces an ensemble neural networks method which divides the entire localization area into regions by clustering the fingerprint database. For each region, a prototype of the received signal strength is determined, and a dedicated Artificial Neural Network (ANN) is trained by using only those fingerprints that belong to this region (cluster).

The Orthogonal Frequency-Division Multiplexing (OFDM) and Multiple Input Multiple Output (MIMO) [21,22] techniques have been widely employed in the IEEE 802.11 standard; thus, the modulated WiFi signals are separated into a number of subchannels in parallel frequencies with different subcarriers and are transmitted concurrently among multipath antennas. A subcarrier is a sideband of a radio frequency carrier wave which is modulated to send additional information. However, RSS data merely shows a coarse value, which neglects the tremendous amount of information contained in plentiful subcarriers and has extreme variability. Compared with RSS values, channel state information (CSI), which can be extracted from new generation wireless network interface cards (NIC), provides more detailed multipath characteristics, including a series of channel measurements depicting the amplitudes and phase distribution of every channel [23,24]; hence, the CSI can be used to replace RSS data to obtain a higher indoor localization accuracy and to overcome the effect of time-varying characteristics. For instance, Reference [25] presents a Fast Orthogonal Search (FOS) algorithm and a Back-Propagation (BP) Neural Network, that can effectively improve the performance in terms of both accuracy and execution time. In addition, the NLOS identification support vector machine (NISVM) and related channel information regression model (RCIRM) are proposed to identify NLOS and mitigate NLOS error in Ref. [26] and obtain better mitigation performance. In addition, it has been detected that the positioning error might be very large in a few cases which might prevent its use in applications with high accuracy positioning requirements. Reference [27] proposes the use of the conditional entropy of a posterior probability distribution as a complementary measure of uncertainty, which can be calculated during localization based on individual sensor measurements, does not require a ground truth, and can be applied to discrete localization algorithms. Reference [28] investigates why large positioning errors occur in Wi-Fi fingerprinting and how to detect them by using the received signal strength intensities.

The present paper shows a novel Random Forest fingerprinting localization (RFFP) method using CSI data to mitigate the limitations of existing machine learning indoor localization methods. Random Forest (RF) is an ensemble classifier that consists of many decision trees (DTs). RF has a number of advantages that make it very suitable for solving many computer vision problems, such as fast training and matching speed, good robustness and stability, high classification accuracy, and good performance for high-dimensional input data [29,30]. The general belief is that CSI fingerprinting performs better in NLOS environments because the multipath provides more channel state information, but experiments reveal that the changing environments yield time-varying CSI data which need data cleaning through the feature selection step in RF. The proposed algorithm includes three steps. First, a subset is randomly extraced from all attributes of CSI values as the training data set to construct a fast decision tree; the size of the RF determines the number of DTs. Second, a root mean squared error (RMSE)-based pruning strategy is applied to reduce the computational complexity as well as overcome the overfitting problem and improve the precision rate of DTs. Third, the votes in the output of every decision tree are counted, and then the most frequently occurring class number is the final classification result of the proposed method. The improved Random Forest-based fingerprinting localization method not only possesses the large number of virtues mentioned previously but also discovers the feature of wireless channel data. Thus, this method can obtain the optimal weights as fingerprints. Meanwhile, the proposed method adopts CSI data to acquire more detailed multipath characteristics than RSS data.

The rest of this paper is organized as follows. A CSI data characteristics analysis is presented in Section 2. The novel Random Forest-based fingerprinting localization method and the RFFP system are detailed in Section 3. Experiments under various conditions are discussed in Section 4. Finally, Section 5 summarizes the whole paper.

## 2. CSI Data Analysis

Wireless signals are separated into numbers of subchannels at parallel frequencies with different subcarriers and transmitted concurrently among multipath antennas in the ubiquitous OFDM systems. However, physical layer (PHY) information was difficult to achieve until the emergence of NICs, such as the Inter Intel WiFi Link (IWL) 5300. Today, CSI data can be suitably extracted by accessing the device driver from a laptop which possesses an IWL 5300, and then better indoor localization accuracy can be obtained due to the outstanding abilities of the anti-attenuation and anti-multipath of CSI data.

Let $\vec{X}$ and $\vec{Y}$ denote the transmitted and received signal vectors. The channel frequency response can be represented as

(1) $$\begin{array}{c} \vec{Y}=\mathrm{CSI}\cdot \vec{X}+\vec{N} \end{array}$$

where $\vec{N}$ represents white Gaussian noise, and CSI represents the channel frequency response.

When IWL 5300 is used in a WiFi channel, this OFDM system consists of 48 subcarriers. Thirty of them are readable as raw binary data through the dedicated device driver. The channel frequency response (CSI${}_{i}$) of subcarrier *i* is a complex value which is defined by

(2) $${\mathrm{CSI}}_{i}=\left|{\mathrm{CSI}}_{i}\right|\exp\left\{j∠{\mathrm{CSI}}_{i}\right\}$$

where $|{\mathrm{CSI}}_{i}|$ and $∠{\mathrm{CSI}}_{i}$ represent the amplitude response and the phase response of subcarrier *i*, respectively.

**Figure 1:**
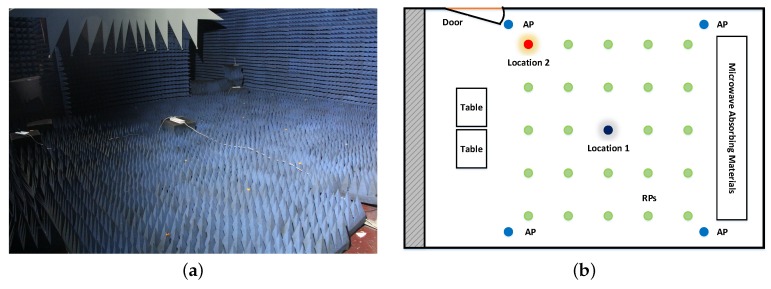
(**a**) Photo of the microwave anechoic chamber. (**b**) Layout of part of the microwave anechoic chamber.

A series of experiments were designed in a microwave anechoic chamber to explore the features of CSI data. The area size is about 160 m${}^{2}$ and four APs were deployed at the corners. All of the APs were working at 2.4 GHz bands. Twenty-five positions, uniformly scattered with one-meter spacing throughout the chamber, were chosen. Figure 1a shows a photo of the microwave anechoic chamber, while the detailed layout and division are shown in Figure 1b.

**Figure 2:**
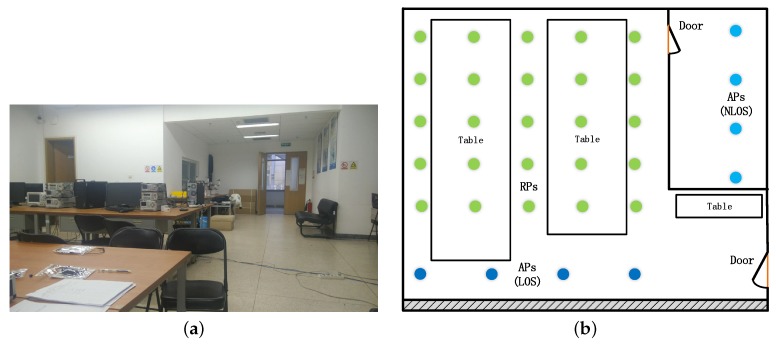
(**a**) Photo of the office. (**b**) Layout of a part of the office.

In addition, a series of experiments were designed in an office to detail the localization performance of RFFP with different system parameters, algorithms, and input datasets at Beihang University. The area of this office is about 80 m${}^{2}$ and four APs were deployed at the different locations to study on the impact of wireless channel circumstances. All of APs were working at 2.4 GHz bands, as before. Twenty-five positions, uniformly scattered with one-meter spacing throughout the office, were chosen. Figure 2a shows the photo of the office, while the detailed layout and division are shown in Figure 2b.

Three signal characteristics of CSI data are present based on the statistical data.

### 2.1. Location Correlation

**Figure 3:**
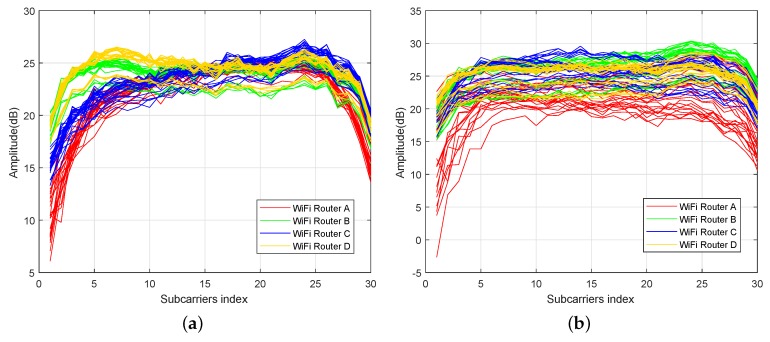
The measured amplitudes of the channel frequency response over 250 s from four different WiFi routers at two different locations of 50 received packets. (**a**) Channel state information (CSI) data at location 1. (**b**) CSI data at location 2.

The CSI data of different APs showed similar amplitude response curves at a fixed location if they were collected by the same antennas. In Figure 3, the amplitude responses of 50 received packets of all 30 subcarriers from four different WiFi routers in two different sampled locations are displayed. Unlike Refs. [24,31] which obtained 90 raw CSI measurements from three antennas of the Intel WiFi link 5300 NIC, each of which collected CSI data from 30 different subcarriers, this paper adopted four single antenna WiFi routers as APs, each of which could provide CSI data with 30 different subcarriers. Thus, it was possible to obtain 120 raw CSI measurements. In both figures, CSIs were measured over 250 s at a fixed location, indicated by a black circle and a red circle respectively in Figure 1b.

The CSI correlation coefficient is introduced into this paper quantitatively analyze the similarity between CSI data collected from different WiFi routers. The correlation coefficient of two random variables is a measure of their linear dependence. If each CSI data has *N* scalar observations, then the Pearson correlation coefficient is defined as [32]:

(3) $$\rho \left(A,B\right)=\frac{1}{N-1}\sum_{i=1}^{N}\left(\frac{A_{i}-\mu_{A}}{\sigma_{A}}\right)\left(\frac{B_{i}-\mu_{B}}{\sigma_{B}}\right)$$

where $\mu_{A}$ and $\sigma_{A}$ are the mean and standard deviation of *A*, respectively, while $\mu_{B}$ and $\sigma_{B}$ are the mean and standard deviation of *B*. The CSI correlation coefficient matrix of CSI data from *N* different WiFi routers is the matrix of correlation coefficients for each pairwise variable combination:

(4) $$R=\left(\begin{array}{cccc} \rho (A,A) & \rho (A,B) & \cdots & \rho (A,N)\\ \rho (B,A) & \rho (B,B) & \cdots & \rho (B,N)\\ \vdots & \vdots & \ddots & \vdots \\ \rho (N,A) & \rho (N,B) & \cdots & \rho (N,N) \end{array}\right).$$

Since *A* and *B*, which represent CSI data from two different sources, are always directly correlated with themselves, the diagonal entries are just 1; thus, ρ(A,B) can indicate the similarity between the two sources of CSI data. The values for ρ(A,B) can range from −1 to 1, with −1 representing a direct, negative correlation, 0 representing no correlation, and 1 representing a direct, positive correlation. The CSI correlation coefficients at location 1 are ρ(A,B)=0.7697, ρ(A,C)=0.7065, ρ(A,D)=0.6886, ρ(B,C)=0.8912, ρ(B,D)=0.8769 and ρ(C,D)=0.8514, respectively. The results indicate that the CSI data of different APs at a fixed location is correlated.

### 2.2. Time-Varying

**Figure 4:**
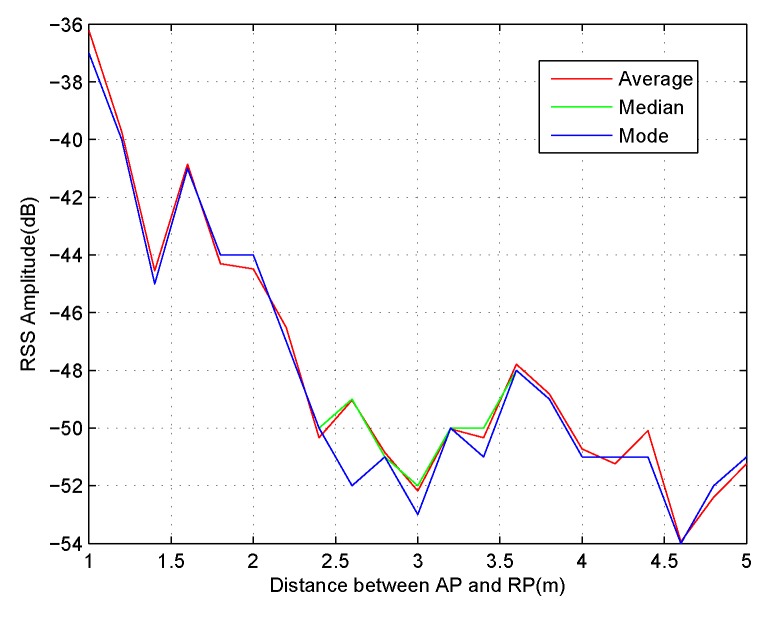
RSS Amplitude values at 21 different locations, which have a distance from 1 to 5 m between AP and RP.

CSI data possess time-varying characteristics, but CSI amplitude responses still exhibit significant stability compared to RSS data. As shown in Figure 3a,b, CSI amplitude curves at a fixed location possess wider value ranges because of the time variation. However, RSS amplitude values show similar characteristics. In Figure 4, the average, median, and mode of the measured amplitude responses of 100 received packets from 21 RPs are displayed and have different distances of 1 to 5 m from AP. The polylines show that the RSS value at a distance of 3.5 m away from AP is greater than that with a 3 m distance from the AP. Considering nothing has changed in the microwave anechoic chamber, this phenomenon occurs just for the higher time variation caused by the current transmit power of APs.

**Figure 5:**
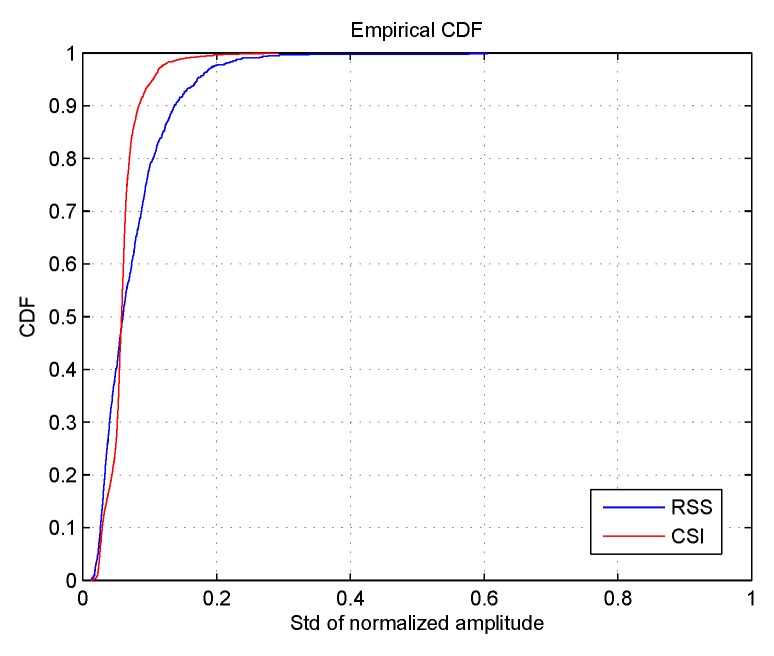
Cumulative distribution function (CDF) of the standard deviations of CSI amplitudes and Received Signal Strength (RSS) values at 25 different locations of more than 2500 received packets.

Figure 5 shows the cumulative distribution function (CDF) of the standard deviations (SD) of normalized CSI and RSS values at 25 different locations of more than 2500 received packets. The maximum and minimum of each subcarrier for CSI data are normalized to 1 and 0, while other values are normalized to proportional numeric values between 0 and 1. As can be seen from this figure, almost 95% of the SDs of normalized CSI amplitude values are lower than 0.1 while less than 80% of the SDs of RSS values can attain the same standard. Thus, CSI amplitude responses exhibit higher stability than RSS values under the exact same experiment conditions. It is worth mentioning that the CSI and RSS data were collected within three days. In fingerprinting, it has been demonstrated that consecutive fingerprints collected within a short period of time are correlated; thus, there are some data dependences.

### 2.3. Incompleteness

As described in the previous section, the CSI data collected by a fixed sampling device in the same location is generally stable; however, a small part of CSI data cannot be acquired in certain places because of the poor CSI signal strength. Therefore, the sensing probability (SP) was introduced as a standard to measure the completeness of CSI data. Assume that there are *n* RPs and *m* APs in the experiment environment. The CSI data acquisition process in the offline training stage can be considered to be a Bernoulli stochastic process. A binary sequence ($B_{j}=\left(b_{1},b_{2},\ldots b_{i},\ldots b_{n}\right)$) can be obtained in each sample of CSI data, where the Boolean variable ($b_{i}$) represents whether the CSI data from the *j*th AP has been received in the *i*th location. The sensing probability (SP) of the *i*th RP to the *j*th AP is given by

(5) $$p\left(b_{i}=1|\omega =\omega_{j}\right)=n\left(b_{i}=1|\omega =\omega_{j}\right)/N\left(b_{i}=1|\omega =\omega_{j}\right)$$

where $n\left(b_{i}=1|\omega =\omega_{j}\right)$ represents the number of times that the *i*th RP can acquire CSI data from the *j*th AP, $\omega_{j}$ represents the *j*th AP, and $N\left(b_{i}=1|\omega =\omega_{j}\right)$ expresses the total number of samples of the *j*th AP that are used to train the model in the offline stage. The sensing probability can be used to indicate CSI signal distribution at different test areas, because CSI signals are lost occasionally during the practical signal collecting process. The packet loss rate ranges from 0% to about 50% at different locations due to poor signal quality and obstacles; therefore, SP can be considered to be a weight value of the CSI signal.

According to these CSI characteristics, the proposed RFFP system adopts node splitting to obtain useful information from collected CSI data. In addition, feature selection is utilized to suppress the multipath effect caused by environmental changes, such as pedestrian movement and item displacement.

## 3. RFFP System

The Random Forest-based fingerprinting localization method and the RFFP system are detailed in this Section. The decision trees which constitute the RF are explained first, and then the construction process of RF is detailed. In the last section of this chapter, the system architecture of RFFP system is introduced. The entire system contains two steps: an offline training stage and an online matching stage.

### 3.1. Proposed Algorithm

The proposed algorithm includes three steps; first, a subset is randomly extracted from all attributes of CSI values as a training data set to construct a fast decision tree; the size of the RF determines the number of DTs. Second, a root mean squared error (RMSE)-based pruning strategy is applied to reduce the computational complexity as well as overcome the overfitting problem and improve the precision rate of DTs. Third, the votes in the output of every decision treeare counted, and then the most frequently occurring class number is the final classification result of the proposed method. The algorithms for creating DTs and constructing the RF are given in the following paragraphs.

#### 3.1.1. Feedback Decision Tree

A decision tree is a rooted tree structure. Each of the non-leaf nodes in a decision tree is a decision node which represents a criterion to divide the cases into two or more subtrees. Each of the leaf nodes represents a cluster of similar cases which are assigned to a single class. A decision tree classifier predicts the class for any unseen case, by going down from the root node to a leaf and using the criteria in the nodes to decide which branch to go [32].

The Feedback Decision Trees (FDT) used in the Random Forest-based fingerprinting localization (RFFP) system are based on the binary recursive partitioning trees. These trees partition the data sample space by using a sequence of binary partitions on individual variables [33]. The root node of the tree comprises the entire sample space ($R^{N}$). The tree nodes which are not split are called leaf nodes and form the final partition of the data sample space. Each non-leaf node splits into two descendent nodes, one on the left and another on the right, according to the splitting criteria of a candidate attribute variable.

Node splitting is the key issue of tree-based classifiers in achieving high accuracy and avoiding overfitting. Much previous attention has focused on the splitting criteria for constructing decision trees and Random Forests [34]. The entropy measure is used to construct the well-known ID3 decision tree [35,36]. The ID3 decision tree learning algorithm calculates the Information Gain (IG) on each attribute (*T*), defined as

(6) $$IG\left(S,T\right)=Entropy\left(S\right)-\sum_{v\in Values\left(T\right)}\frac{\left|S_{v}\right|}{\left|S\right|}Entropy\left(S_{v}\right)$$

where *S* is the total input space, and $S_{v}$ is the subset of *S* for which attribute *T* has a value *v*. The Entropy(S) over *s* classes is given by $\sum_{i=1}^{s}-p_{i}\log_{2}\left(p_{i}\right)$, where $p_{i}$ represents the probability of class *i*. The attribute with the highest information gain is chosen as the root node of the tree. Next, a new decision tree is recursively constructed over each value of the attribute with the highest information gain using the training subspace ($S-S_{B}$). A leaf-node or a decision-node is formed when all the instances within the available training subspace are from the same class.

An improved version, namely the C4.5 tree, was proposed in which the normalized information gain is used as the splitting criterion. C4.5 enumerates all the possible tests and chooses the best one. By default, C4.5 uses the information gain ratio to determine how good a test is:

(7) $$GainRatio\left(S,T\right)=\frac{Gain\left(S,T\right)}{SplitInformation\left(S,T\right)}$$

where Gain(S,T) is the information gain of the test, and SplitInformation(S,T) is the amount of information of the split caused by the test which is given by

(8) $$SplitInformation\left(S,T\right)=-\sum_{i=1}^{s}\frac{\left|S_{i}\right|}{\left|S\right|}\log_{2}\frac{\left|S_{i}\right|}{\left|S\right|}$$

where $S_{1}$, …, $S_{s}$ are subsets of *S* corresponding to cases with different known values.

The Classification and Regression Tree (CART) is one of the classification algorithms and was proposed by Breiman [37] in 1984. The CART involves the construction of a decision binary tree structure based on the training data set consisting of features and target variables through iterative analysis [38]. The CART algorithm employs the Gini Index as the criteria for choosing the best characteristic variables [39]. The Gini Index of set *S* is defined in the following text.

In FDT, the information gain ratio defined in Equation (7) is used as the criterion for the splitting and reduced-error pruning strategy with feedback applied to the tree building phase. The pseudocode for the FDT building phase with multiple packets is given in Algorithm 1. Firstly, *m* packet receptions each with 120 CSI values for each of *N* training locations are collected through IWL 5300 as the input dataset. Let *X* be the input dataset and a Feedback Decision Tree (*T*) be an output. In fact, the building phase includes three steps: the pretraining and initialization process in lines 1–2, the node splitting and iteration-stopping criterion in lines 3–13, and the pruning and feedback strategy in lines 14–17. For pretraining and initialization, *k* fold cross-validation is applied to choose subdatasets for training, and then a feature selection algorithm is employed in order to generate a new training set, and an empty FDT is initialized.

Because the feature points of datasets are usually in high-dimensional space, direct estimation of the distribution is still not a good practice. Feature selection [40] is a significant step of various LBSs and pattern recognition problems. Likewise, this method is adopted in the building phase of FDT to identify suitable features. In order to produce a reduced CSI dataset, various feature selection approaches along with various search methods are available. Filter, Forward/Backward search, and Wrapper are generally regarded as feature selection methods, and the results obtained from these methods differ in accuracy as well as in processing time. The feature selection method is used to identify the suitable features by means of various assessment functions, namely, the classifier error rate, dependency, information, consistency, and distance [41]. FDT adopts feature selection to detect dependencies between features in datasets in order to conclude whether the selected subset feature is optimal or not.

The mutual information was utilized to measure the correlation coefficient between features $x_{i}$ and label *y* during filter feature selection in this study. The mutual information is given by

(9) $$MI\left(x_{i},y\right)=\sum_{x_{i}}\sum_{y}p\left(x_{i},y\right)\log\frac{p\left(x_{i},y\right)}{p\left(x_{i}\right)p\left(y\right)}$$

where $p\left(x_{i},y\right)$, $p\left(x_{i}\right)$ and $p\left(y\right)$ can be calculated from dataset.

**Algorithm 1.** Building phase of the Feedback Decision Tree
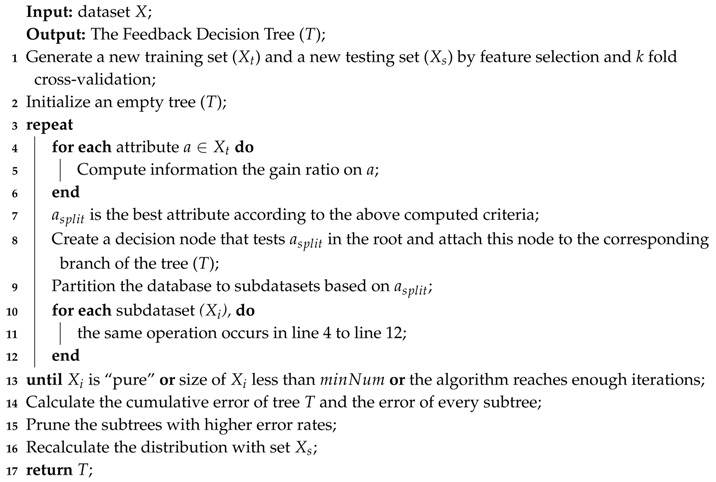


Once the new training set ($X_{t}$) has been completely generated, the information gain ratio of all attributes from $X_{t}$ is calculated. The best attribute with the highest information gain ratio is called $a_{split}$, so a decision node can be produced and attached to the corresponding branch of the tree (*T*). Hereafter, some subdatasets are partitioned from the $X_{t}$ based on $a_{split}$, and each of them is included in the repetitive cycle process until the ceasation condition is fulfilled. Significantly, the stopping criteria in this study consist of three situations: (1) the subset is “pure”, in other words, there is not any other category in this subset; (2) the size of this subset is less than minNum, which can be set to empirical values; and (3) the algorithm reaches enough iterations in order to overcome overfitting problem as well as to reduce the complexity.

Furthermore, the dimension of features with the smallest generalization error can be calculated though cross-validation. Ten-fold cross-validation is utilized in the RFFP system. Specifically, the original CSI data is randomly partitioned into 10 equal-sized subsamples. One single subsample of all the subsamples is retained as the validation data to test the model, and the remaining nine subsamples are used as training data. This process is repeated 10 times; thus, an average localization accuracy can be obtained to give an estimate of the model’s predictive performance. Thereafter, the pruning step with reduced-error pruning strategy and a feedback process which recalculates the distribution with dataset $X_{s}$ tailor the FDT to reserve indispensable nodes. The pruning step calculates the cumulative error of tree *T* and the error of every subtree. If the cumulative error of a subtree is greater than tree *T*, this node will be replaced with its most popular class. Sometimes, more than one node reaches the pruning condition, but the best method is probably not to replace all nodes. Therefore, the best FDT which is eventually adopted to construct the RF is chosen by generalized accuracy, as measured by a training set through the feedback process. Since CSI data possess obvious time dependence characteristics, it is essential to recalculate the distribution with a new incoming dataset timely.

#### 3.1.2. Random Forest

In the construction stage of Random Forest, an ensemble method is adopted to accurately classify test data. The Bootstrap Aggregation method which was proposed by Breiman [42] was applied in this study not only to achieve a higher accuracy rate but also to obtain higher system robustness. The pseudocode for the RF construction phase with multiple CSI packets is shown in Algorithm 2. The input datasets are m packet receptions each with n CSI values from each of *N* training locations, collected through IWL 5300. Another input is the size of the forest (*S*) which determines how many FDTs will be built. Let *X* be the input dataset; meanwhile, a Random Forest (*F*) is the output. The construction phase of Random Forest can be divided into three steps: attribute selection and datasets initialization in lines 1–5, FDT training in lines 6–8, and FDT combination and RF generation in lines 9–10. First, the new training dataset and testing dataset for every FDT are generated using the Bootstrap Aggregation algorithm. More specifically, for every training set ($X_{t}$) which is collected by 10-fold cross-validation, bagging generates *S* new training sets ($X_{t}^{i}$) by sampling from $X_{t}$ uniformly and with replacement. The same process is applied to generate new testing sets ($X_{s}^{i}$). By sampling with replacement, some observations may be repeated in each $X_{t}^{i}$. However, a single CSI data will not appear in both the training set and the testing set because of the 10-fold cross-validation. Then, the sensing probability of each dataset is calculated and added into the corresponding dataset. For every branch of a FDT, *K* different attributes are utilized randomly in node splitting so that the feature of CSI data in 120 subchannels can be analyzed meticulously. *K* usually sets the square root of the size of the training dataset which is n+1. It is worth mentioning that those FDTs do not experience pruning in order to allow their features to be retained as much as possible. In the end, *S* Feedback Decision Trees are combined under the thought of voting algorithm.

**Algorithm 2.** Random Forest construction with Feedback Decision Tree
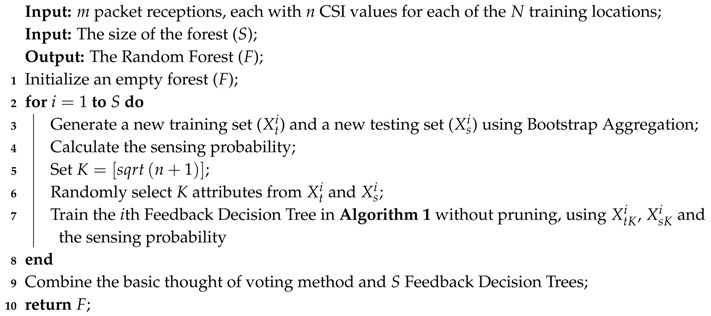


The Bootstrap Aggregation algorithm generates a new individual training set which is called the bootstrap sample from the original data for every FDT set input . Therefore, different Feedback Decision Trees can be established with an individual training set. Random Forest combines those FDTs through voting so that the output of RF is the class predicted most repeatedly by its subclassifiers.

### 3.2. RFFP System Architecture

**Figure 6:**
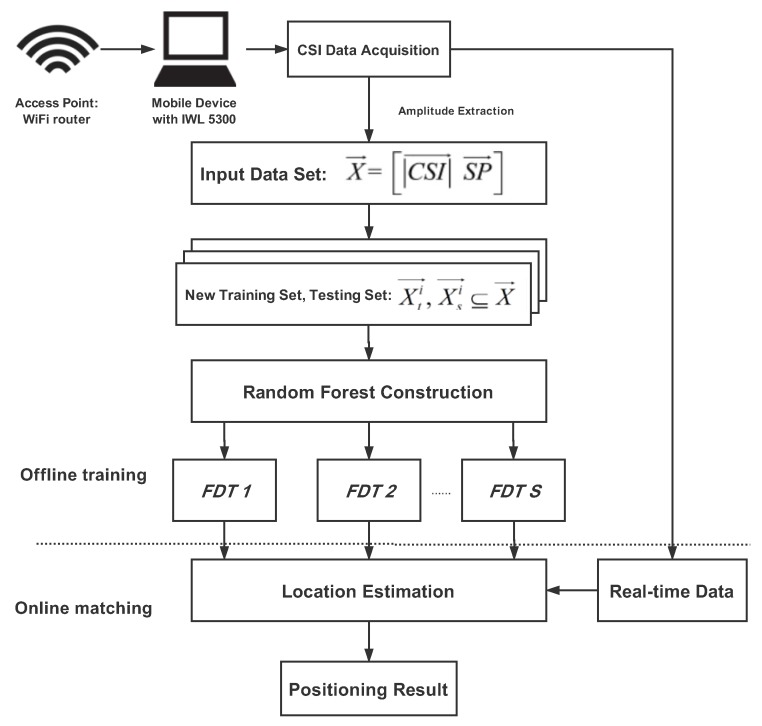
Random Forest fingerprinting localization (RFFP) system architecture.

The system architecture of the RFFP system is shown in Figure 6, where $\left|\vec{CSI}\right|$ is the amplitude response of CSI data and $\vec{SP}$ is the sensing probability. $\vec{X_{t}^{i}}$ and $\vec{X_{s}^{i}}$, which are generated by *k* fold cross-validation, are subsets of the input data set ($\vec{X}$). Actually, four WiFi routers were utilized as access points in this study as well as a laptop with an IWL 5300 NIC as the mobile device. The IWL 5300 NIC can read 30 different subcarriers from each WiFi router, thus it was possible to acquire 120 CSI values simultaneously from four individual APs in every RP. Unlike RSS which reflects the amplitude of overlapped multipath signals, CSI measurements from 120 subcarriers of four individual WiFi routers were used in this study to exploit the features in physical layer thoroughly. The entire system contained two steps: an offline training stage, and an online matching stage.

The offline training stage can be divided into three parts: CSI data acquisition, data preprocessing and Random Forest construction.

(1) CSI data acquisition: The RFFP system gathers untreated CSI data from 25 Receiver Points and then stores it in the original database. Every received packet contains the complete information from four WiFi routers, each of which contains 30 subcarriers. Those packets are utilized to generate fingerprints based on the amplitude features of CSI data and the sensing possibilities of different routers.

(2) Data preprocessing: New training sets and testing sets are generated through the Bootstrap Aggregation algorithm and *k* fold cross-validation as well as being used to building FDTs.

(3) Random Forest construction: FDTs without pruning are established for each training set. These FDTs are stored as improved fingerprints and utilized in location estimation.

**Figure 7:**
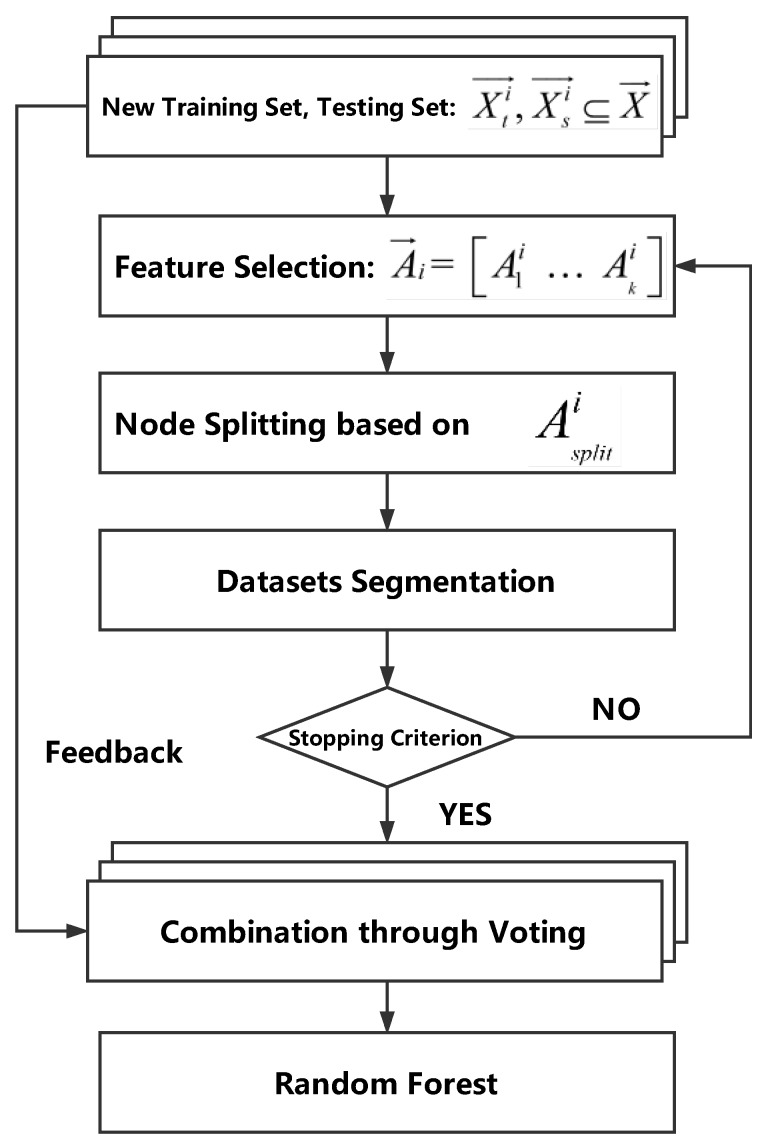
Technical details in Random Forest construction.

Figure 7 shows the technical details of the Random Forest construction phase. In fact, the size of this fingerprint database merely depends on the size of RF, so that only a handful of information need to be stored in order to estimate the location.

In the online matching stage, the real-time CSI data which is collected by a mobile device can be input into RF; thus, positioning result can be estimated through the voting method.

Compared with other algorithms, like KNN and WKNN, which have to store all of the CSI data in the fingerprint database and piecemeal one-by-one comparisons in the online matching stage, the RFFP system only needs to keep the model trained in offline training stage and carry out the node splitting process in the online matching stage. Specifically, each transmit-receive antenna pair provides 30 subcarrier channels and a single package is about 5 KB, thus a CSI database with *n* RPs, *m* APs and *p* packets should be nmp×5 KB. On the other hand, a single FDT is about 100 KB; thus, a RFFP with *t* FDTs should be t×100 KB. It is worth mentioning that *t* generally chooses 100 to approach optimum performance, while increases in *m* and *n* lead to larger storage. Therefore, RFFP system has the advantages of a low workload and less storage consumption.

## 4. Experiments and Discussion

The experiments conducted under various conditions are discussed in this section. After introducing the experiment setting, three different impacts are discussed to evaluate the localization precision of RFFP system: (1) the impact of wireless channel circumstances; (2) the impact of the wireless channel environment; and (3) impact of the input datasets. In addition, the effects of different hyperparameters are shown at end of this section: (1) the impact of maximum depths of FDT; (2) the impact of the number of FDTs in RFFP; and (3) the impact of the number of features.

### 4.1. Experiment Setting

Since different experimental conditions strongly affect the location system’s performance, detailed directions of experimental environment setting are meaningful. This study conducted experiments in a microwave anechoic chamber and an office at Beihang University. The environments where the experiments were performed are shown in Figure 1a and Figure 2a. In the meantime, the circles in Figure 1b and Figure 2b display the 25 locations of the Receiver Points . The area size of a microwave anechoic chamber is 160 m${}^{2}$, while four APs, each with one transmitted antenna, are deployed at the corners. It is worth mentioning that there are no routers in the chamber, but the authors deployed four routers next to the wall at a height of about 2.5 m, as shown in Figure 1. The antennas of the APs were vertically positioned. In addition, the area of this office is about 80 m${}^{2}$ and four APs were deployed at different locations to study the impact of the transmission environment. In LOS scenarios, the APs were deployed on the tables of 0.8 m height. APs were deployed at a separate room in the NLOS scenario; thus, over 50% of the Fresnel zone was blocked while APs and RPs could not see each other. It is believed that signals are impeded in this situation, and we called it the NLOS scenario. The APs were about 0.5 m from the wall and rested on counters of 2.1 m height in this scenario. In both scenarios, the directions of antennas did not need any special setup, as before. Twenty-five positions, uniformly scattered with one-meter spacing in the chamber and the office, were chosen. Considering that users could stand anywhere in the test area, a positioning result which is given by classification based localization system could possess an additional location error. In this system, the additional location error could range from 0 m to $\frac{\sqrt{2}}{2}$ m.

Two major instruments were implemented in this study: the APs, which were four TP-Link routers, and a mobile device, which was a Lenovo laptop with IWL 5300 NIC installed. At the mobile terminal, the Linux 802.11n driver and the Ubuntu Linux system were installed; thus, wireless signals from APs could be received through the IWL 5300 NIC and then stored in the laptop. Unlike Ref. [31], the TL-WR742N routers employed in this study had merely one transmitted antenna, so that only one single transmitter–receiver antenna pair for each AP was readable. The mobile device measured CSI values through the receiver antenna, and thus there were four transmitter-receiver pairs available for feature collection. Each antenna pair gathered a channel state of 30 subcarrier channels; therefore, a total of 120 subcarriers were utilized in this study. After modifying the kernel of the Linux 802.11n driver, raw CSI data was transferred to the mobile device and processed with the CSI data acquisition program. The specific steps were as follows: (1) The prerequisites were installation of the build tools, the Linux development headers, and the Git client; (2) build and install the modified wireless driver. In this step, the modified driver was built for the existing kernel, and then, the modified driver was installed into the module updates directory; (3) install the modified firmware. Specifically, the CSI Tool supplementary material was obtained, any existing firmware for Intel Wi-Fi Link 5000 Series adapters was relocated, and the modified firmware was installed; (4) the Userspace Logging Tool was built; and (5) an 802.11n access point was connected to begin logging CSI to a file. At the access points, WiFi routers took charge of consecutively transmitting CSI packets to the mobile terminal which required a localization and positioning service. The Linux ping command was employed to generate the request in order to periodically obtain CSI packets returned by routers. More specifically, the laptop sent pings consistently by a program designed by the authors at a rate of 10 times per second, and then, the current router returned a CSI packet each time it received a ping. As soon as 50 packets of a single router had been collected, the Java program sent a reconnection command to connect another router which had not been utilized at this receiver location. The program repeated this process in order to gather all CSI features from four routers at every selected receiver location. As a consequence, the original dataset with 25 locations, each with 50 CSI packets, was stored in a 120-dimensional matrix.

The raw CSI data which was recorded in the 120-dimensional matrix was further processed. First and foremost, the sensing probability was calculated through the proposed algorithm as well as stored in the 124-dimensional matrix, which was composed of the original CSI data submatrix and sensing probability submatrix. Second, the Bootstrap Aggregation method was applied to generate individual training sets and testing sets to build each FDT. Third, all new datasets were used for fingerprinting and position estimating. The Out-Of-Bag (OOB) samples which were not chosen for training FDT were used to calculate an unbiased estimation of the generalization error, named the Out-Of-Bag-Error (OOBE) [43]. The OOBE is the average error for each dataset (S) calculated using predictions from the trees that do not contain S in their respective bootstrap samples. The OOB estimate is as accurate as using a test set of the same size as the training set; therefore, this allows the RFFP to be fit and validated whilst being trained. In addition, the true positive rate (TP Rate), false positive rate (FP Rate), precision, accuracy, and F-Measure were introduced in this article to show the significant performance of the algorithms. These properties are defined as follows:

(10) $$TP\;Rate=\frac{True\;Positive}{True\;Positive+False\;Negative}$$

(11) $$FP\;Rate=\frac{False\;Positive}{True\;Negative+False\;Positive}$$

(12) $$Precision=\frac{True\;Positive}{True\;Positive+False\;Positive}$$

(13) $$Accuracy=\frac{True\;Positive+True\;Negative}{Positive+Negative}$$

(14) $$\mathrm{F-Measure}=\frac{2\cdot Precision\cdot TP\;Rate}{Precision+TP\;Rate}$$

For every location which is regarded as a single class in RFFP, the true positive stands for the sample of this location that is properly classified, while a false negative is an error in which a sample of other locations is identified at this location. Actually, true negatives are meaningless in an n-class classifier; therefore, this value is 0. Thus, the number of actual negative values is equal to the number of false positives, and the number of actual positive values is equal to the number of true positives plus false negatives in Equation (13). The OOBE, TP Rate, FP Rate, precision, accuracy, and F-Measure together form the evaluation system.

### 4.2. Localization Precision

The performance of an indoor positioning system cannot be separated from environmental factors; thus, a series of experiments were done to evaluate the localization precision of RFFP system under different circumstances. Three different impacts are discussed in this section: (1) the impact of wireless channel circumstances; (2) the impact of the wireless channel environment; and (3) the impact of input datasets.

#### 4.2.1. Impact of Wireless Channel Circumstances

To evaluate the performance of RFFP system under different wireless channel circumstances, different algorithms, including KNN, WKNN, ensemble of BPNNs, J48 Tree, CART Tree, REPTree and RFFP, are utilized with exactly the same input datasets. The Mean Absolute Error (MAE) and classification accuracy between different algorithms and input data are presented in Table 1 and Table 2. The ensemble classification neural network method was introduced in Ref. [44] to deal with noisy WiFi signal strength, and experiments show this method is capable of replacing GPS for the indoor environment. The ensemble of BPNNs consists following steps:

(1) Construct a bootstrap sample by randomly sampling with replacement n times from original data.

(2) Repeat step (1) for K times when K is large.

(3) Construct K simple neural networks.

K was chosen to be 50 in Ref. [44] and this section, and in particular, BPNNs were aggregated by a voting procedure to get a classification result. For each training instance, the BPNN first made a prediction, measured the error, and then went through each layer in reverse to measure the error contribution from each connection, and finally, slightly tweaked the connection weights to reduce the error.

**Figure 8:**
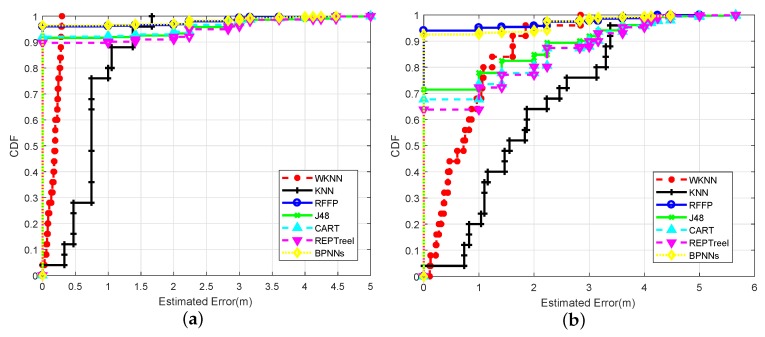
(**a**) CDFs of localization errors with different algorithms in the microwave anechoic chamber. (**b**) CDF of localization errors with different algorithms in the office (LOS).

The CDFs of localization errors with different algorithms in the microwave anechoic chamber are shown in Figure 8a. As can be seen from this figure, the RFFP system achieved excellent performance with an accuracy of 96.04%. In other words, more than 95% of the test data acquired a localization result with no error. This categorization accuracy was significantly higher than the J48 Tree with 91.64%, the CART Tree with 92.08%, and the REP Tree with 89.68%, but lower than ensemble of BPNNs with 96.36%. In addition, the MAE of RFFP was 0.1033 m which could be further decreased by tracking the trajectory. WKNN achieved an acceptable result with a MAE of 0.1767 m and KNN obtained a barely satisfactory location accuracy with a MAE of 0.7608 m. Although the microwave anechoic chamber reduced the multipath effect so that all of three algorithms acquired favorable results with MAEs under 1 m, it is remarkable that RFFP achieved a significant improvement over the WKNN and KNN algorithms.

Figure 8b displays the CDFs of estimated errors with six different algorithms in the office. The RFFP system performed well with an accuracy of 93.12%. This categorization accuracy was still higher than ensemble of BPNNs with 92.52%, the J48 Tree with 71.44%, the CART Tree with 67.68% and the REP Tree with 63.76%. On the other hand, the MAE of RFFP was about 0.17 m which could be further decreased by tracking the trajectory. In contrast, WKNN had a MAE of 0.8164 m, and KNN obtained a barely satisfactory location accuracy with a MAE of 1.8421 m. It follows that performance degradation was shown in all the algorithms when the wireless channel circumstances changed. Meanwhile, the localization performance of RFFP declined the least among the six algorithms. Specifically, the localization accuracy levels of J48, CART, REPTree, ensemble of BPNNs, and RFFP decreased by 20.20%, 24.40%, 25.92%, 3.84%, and 2.92%, respectively. In addition, the MAEs of KNN, WKNN, and RFFP increased by 1.0813 m, 0.6397 m, and 0.0675 m, respectively. Based on the above, one can draw the conclusion that RFFP is more able to adjust to the usual office environment.

In the microwave anechoic chamber, the experimental results indicated that the RFFP system not only achieved improvements over KNN and WKNN in reducing MAE by 86.4% and 41.5%, but also outperformed REPTree, CART, and J48 in terms of localization accuracy by 6.36%, 3.96%, and 4.40%, respectively. In the office, the RFFP system still showed a better performance. The MAEs of RFFP, KNN, and WKNN were 0.1708 m, 1.8421 m, and 0.8164 m, respectively. Meanwhile, the localization accuracy in the office of the RFFP system was higher than the ensemble of BPNNs, REPTree, CART, and J48 by 0.6%, 29.36%, 25.44%, and 21.68%, respectively. Extensive experimental results showed that the RFFP system is robust against the multipath fading caused by the numerous obstacles in the office.

The TP Rate, FP Rate, precision and F-Measure of the RFFP system were 0.931, 0.003, 0.934, and 0.931, respectively, that is, true positive specimens with precise judgments accounted for greater than 93% of both the total true positive specimens and all positive examples. The detailed statistics indicated that the RFFP system is capable of achieving robust localization performance and considerably reducing the estimate error compared with KNN and WKNN. This article further discusses the various effects of the RFFP system, including the different input datasets and system parameters which contain a number of FDTs in Random Forest, the max depth of a single FDT, and the quantity of features applied at the feature selection stage of RFFP.

#### 4.2.2. Impact of Channel Environment

**Figure 9:**
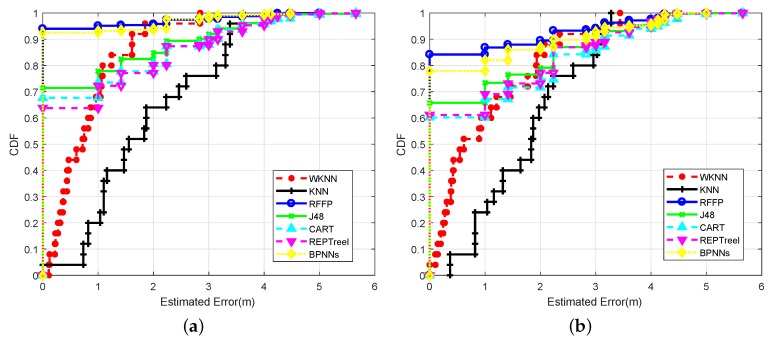
(**a**) CDFs of localization errors with different algorithms in LOS scenarios. (**b**) CDFs of localization errors with different algorithms in NLOS scenarios.

To research the effects of different sight environments on the RFFP system’s performance, two individual experiments were conducted: (1) four WiFi routers were placed under the LOS scenario as shown in Figure 2b; and (2) four WiFi routers were placed under the NLOS scenario. Figure 9 shows the impact of the sight environment on the performance of the proposed RFFP. From Figure 9a,b, we can observe that nearly all algorithms performed better for the LOS scenario, except for KNN. More detailed localization performances are shown in Table 1 and Table 2.

The CDFs of localization errors with different algorithms in the office under the LOS scenario are shown in Figure 9a. As can be seen from this figure, the RFFP system achieved the best performance with an accuracy of 93.12%; this categorization accuracy is higher than ensemble of BPNNs with 92.52%, the J48 Tree with 71.44%, the CART Tree with 67.68%, and the REP Tree with 63.76%. In addition, the MAE of RFFP was 0.1708 m in this case. WKNN had a MAE of 0.8164 m, and KNN obtained a barely satisfactory location accuracy with a MAE of 1.8421 m.

Figure 9b displays the CDFs of estimated errors with six different algorithms in the office under the NLOS scenario. The RFFP system still performed the best with an accuracy of 84.18%. This categorization accuracy was still higher than the ensemble of BPNNs with 77.88%, the J48 Tree with 65.74%, the CART Tree with 60.20% and the REP Tree with 61.13%. On the other hand, the MAE of RFFP was 0.4033 m. In contrast, the WKNN had a MAE of 1.0517 m and KNN obtained a location accuracy with a MAE of 1.7782 m, which was slightly better than the LOS case. It follows that performance degradation was shown in almost all of the algorithms under the NLOS scenario. Meanwhile, the localization performance of RFFP continued to be the best. The reason why the performances of all algorithms declined is that CSI signals were lost occasionally in particular data collecting processes. However, when an imperfect CSI packet enters the RFFP system, the node splitting process can immediately find the most important parts of data and then train the model or obtain localization result. Based on the above, we can assume that RFFP provides the best performance in the usual office environment.

#### 4.2.3. Impact of Input Datasets

To evaluate the effects of different types of input data on RFFP system performance, two individual experiments were conducted: (1) RFFP with 120-dimensional CSI data from four different WiFi routers as input data compared with RSSI data from identical routers; and (2) RFFP with CSI data range from 30 to 120 values from differing numbers of APs. In addition, all of other system parameters were kept the same as before, and the impacts of those parameters are discussed in the sections below.

As shown in Table 1, the KNN algorithm with RSSI data as input achieved an estimated error of around 1.7 m, while the WKNN algorithm with the same input had a mean localization error of about 1.13 m, whereas the CSI input datasets had reduced MAEs of 0.76 m and 0.18 m, respectively, for KNN and WKNN. Therefore, CSI can be considered a more effective input than RSSI.

To evaluate the impentact of the number of transmitter-receiver antenna pairs which is highly dependent on the number of APs, a specific experiment was conducted by utilizing different numbers of WiFi routers to evaluate the impact on not only the localization accuracy, but also, other performance measures, including MAEs, OOBE, the TP Rate, the FP Rate, precision, and the F-measure. In addition, other system parameters remained unchanged in this controlled experiment.

Table 3 shows the mean localization error, classification accuracy, TP Rate, FP Rate, precision, and F-measure of these experiments with different numbers of APs. Every utilized AP in RFFP only had one transmitting antenna so that merely 30 CSI values could be collected from it. In addition, in order to eliminate the effect of location of APs, all the APs combinations were utilized to acquire the average localization result. As the number of APs increased, the upwards trend of the classification accuracy became slower while the mean positioning error dropped significantly. Furthermore, the TP Rate, precision and F-Measure showed obvious upward tendencies. All of those assessment parameters had sharp variations when the number of APs increased from 1 to 3; nevertheless, this variation became gentler when the number of APs further increased. More specifically, the classification accuracy, TP Rate, precision, and F-measure increased by 14.64%, 14.6%, 14.4%, and 14.8% when the number of APs augmented from 1 to 2. However, those assessment parameters merely increased to above 2.5% and 0.6%, while the number of APs changed from 2 to 3 and 3 to 4, respectively. In addition, the OOBE and FP rate showed the same characteristics when the number of APs changed, except for the downwards trend which indicates positioning errors are reduced as the amount of APs increases. As a result, we came to the conclusion that at least two transmitter-receiver antenna pairs which possess 60 CSI values can satisfy the requirements of high precision positioning; nevertheless, continuing to increase the number of antenna pairs or APs brought little direct benefit. On the other hand, a great number of WiFi routers have more than one antenna so that more CSI values can be obtained from added subcarriers, which means increasing the number of APs is not necessary for achieving greater CSI values.

Although the four-AP RFFP system can achieve a lower localization error, it requires a bit more time to train the 120-dimensional CSI data in the RF construction. As shown in Table 3, each additional AP brings about one more second for building time of RFFP. Considering that the construction stage only occurs once in the entire system, this time consumption is regarded as both rewarding and acceptable. Therefore, this article proposes the maximal utilization of existing facilities within a reasonable scope.

### 4.3. Effects of Different Hyperparameters

An ensemble classifier like RF processes some hyperparameters which are correlated with classification accuracy. Thus, the effects of different hyperparameters are shown in this section: (1) the impact of the maximum depth of FDT; (2) the impact of the number of FDTs in RFFP; and (3) the impact of the number of features.

#### 4.3.1. Impact of Maximum Depths of FDT

**Figure 10:**
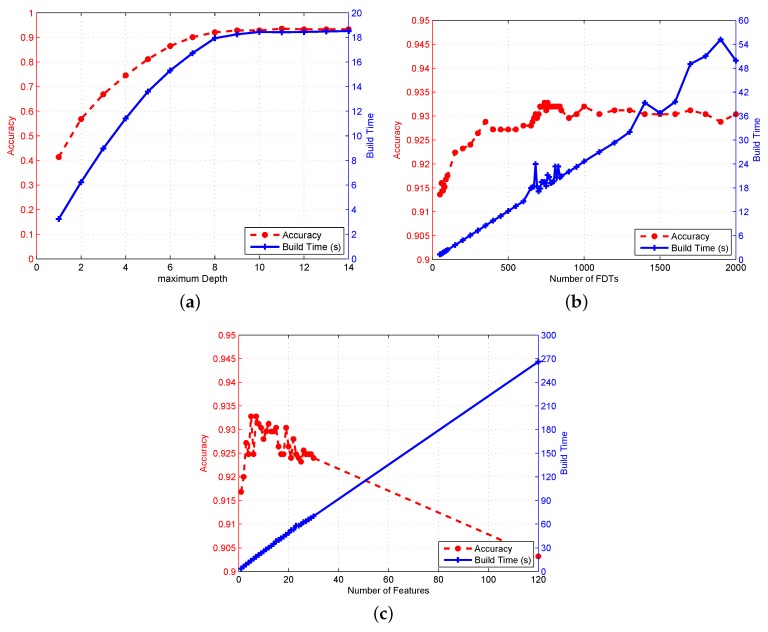
The classification accuracy and build time of RFFP with different system parameters. (**a**) with different max depths of FDT. (**b**) with different number of FDTs utilized in RFFP. (**c**) with different number of features chosen for feature selection stage in RFFP.

To evaluate the effect of max depths of FDT on RFFP system localization performance, a specific experiment involving setting different iteration-stopping criteria was designed. In FDT, the splitting process is normally executed repeatedly until one of the following conditions is reached: (1) subsets are “pure”; or (2) the size of subset is less than minNum; or (3) the FDT reaches enough iterations. The maximum depth that is totally dependent on the iterations is a hyperparameter. Consequently, the iterations of FDT were set as different amounts, and the results are shown in Figure 10a.

In cases where there is a limited quantity of iterations, node splitting may be terminated early. The restricted FDT brings not only a faster processing speed but also a capacity to overcome overfitting. Nevertheless, excessive restraints on the maximum depth are also inherently problematic. In detail, an insufficient number of iterations is unable to split the attributes of input datasets deeply and meticulously, which will result in classification accuracy reduction.

As can be seen from the figure, localization accuracy had a sharply increasing tendency when the maximum depth of FDT changed from 1 to 8, and this curve changed smoothly when the maximum depth exceeded 10. It is worth mentioning that the time spent on the RF construction stage showed this similar sharp trend; therefore, it is considered that every second spent on the increase in maximum depth is meritorious. To be specific, RFFP with a depth of 8 obtained an accuracy surpassing 92%, while another RF with a depth of 2 only had an accuracy rate of 41.36%. On the other hand, the time consumed to establish the above two models was 3.24 s and 17.93 s, respectively.

#### 4.3.2. Impact of the Number of FDTs in RFFP

Since the size of RF changes as the number of FDTs utilized in RFFP varies, this paper examined the impact of varying the number of FDTs in the RFFP system through following a specific experiment. First, the maximum depth of FDT was not set to any value in order to generate each FDT in as intact of a state as possible. Then, a number of individual RFs were constructed with different amounts of FDTs to evaluate the effect of the size of RF on RFFP system. In addition, other hyperparameters, such as the maximum depth and feature numbers remained unchanged at this point, while the RFFP system architecture was invariant which means the input datasets and the locations of APs and RPs were essentially the same. At last, the amount of time spent on training model compared with the localization accuracy is presented in Figure 10b.

Unlike the maximum depth of FDT, the impact of number of FDTs in RFFP on the construction time was practically linear. It is reasonable, as well as acceptable, to make the conclusion that every FDT costs almost exactly the same amount of time in the training stage; however, the practical time consumption in the actual environment is normally influenced by the operational status of CPU or GPU. That is probably why the margin fluctuation of curve appears in Figure 10b. From another perspective, the effect on positioning accuracy is worthy of attention. The RFFP with 50 trees obtained a satisfactory performance which required merely 1.31 s to train the model and achieved a localization accuracy of 91.36%. Nevertheless, the RFFP with 2000 trees did no tachieve an outstanding performance, or more accurately, the time consumption of establishing this model was 49.94 s, which was about 38 times longer than before. However, the classified accuracy was 93.04%, which achieved only a 1.68% increase compared to the 50-tree system. Moreover, the best result appeared in the 740-tree RFFP system with a classification accuracy of 93.28%. Therefore, regarding the overfitting problem and time consumption, it is recommended that the number of FDTs in RFFP is an empirical value of 100 or other integer that fits the performance of CPU or GPU to guarantee the instantaneity of RFFP system.

#### 4.3.3. Impact of the Number of Features

To study the effect of the number of features chosen for feature selection stage in RFFP, a specialized experiment which utilized different numbers of features was conducted to estimate the influence on both location precision and execution time. As previously mentioned, RFFP collected 120 CSI values for each of the 25 training locations as the input datasets. Afterwards, *k* fold cross-validation and feature selection were utilized to choose the subsets for training. What is worth mentioning is that the central theme of feature selection in RF is mining characteristics of input datasets as much as possible. More specifically, extracting fewer features can expose a specific attribute, while selecting major features can reduce the impact of specific abnormal data.

As displayed in Figure 10c, the optimum imitative straight line of elapsed time overhead on establishing training model is pretty meaningful. Since the number of features is proportional to the training time, it is reasonable to concentrate on the relation between localization performance and the number of selected features. When a single feature was selected to train a RF, the accuracy rate of classification was 91.68%, while the elapsed time was 3.7 s. As selected attributes increased from 1 to 12, there was an upward trend of positioning accuracy which ranged from 91.68% to 93.12%. After that, adding features resulted in a declining tendency because the attributes with more dominant characteristics conceal and overshadow other attributes. Consequently, the RFFP with all 120 attributes in feature selection achieved an accuracy of only 90.32% but required 266.06 s for model training. Thus, regarding the localization performance and time consumption, it is recommended that the number of features utilized in the feature selection of RFFP is an empirical value of the square root of the sum of input attributes.

## 5. Conclusions

In this paper, we tackled the problem of CSI-based localization system by the RFFP, which is a Random Forest-based indoor fingerprinting system that utilizes FDT. The CSI information in the physical layer of all subcarriers from four different WiFi routers was collected and utilized in the RFFP model training stage after calculating the sensing probability and selecting features. Furthermore, a series of specific experiments were conducted in a microwave anechoic chamber and an office to detail the localization performance of RFFP with different system parameters, algorithms, and input datasets. Extensive experimental results showed that with a measurement resolution of 1 m, the proposed RFFP achieved MAEs of 0.1033 m, 0.1708 m, and 0.4033 m in the microwave anechoic chamber, and the office under LOS and NLOS scenarios, respectively. In the microwave anechoic chamber, experimental results indicated that the RFFP system not only improves KNN and WKNN by reducing the MAEs by 86.4% and 41.5%, but also outperforms REPTree, CART, and J48 in terms of the localization accuracy by 6.36%, 3.96%, and 4.40%, respectively. In the office under the LOS scenario, the RFFP system still showed a better performance. The MAEs of RFFP, KNN, and WKNN were 0.1708 m, 1.8421 m, and 0.8164 m, respectively. Meanwhile, the localization accuracy of the RFFP system in the office was higher than the ensemble of BPNNs, REPTree, CART, and J48 by 0.6%, 29.36%, 25.44%, and 21.68%, respectively. In the office under the NLOS scenario, the MAEs of RFFP, KNN, and WKNN were 0.4033 m, 1.7782 m, and 1.0517 m, respectively. Meanwhile, the localization accuracy of the RFFP system was higher than the ensemble of BPNNs, REPTree, CART, and J48 by 6.3%, 23.05%, 23.98%, and 18.44%, respectively. Extensive experimental results showed that the RFFP system offers outstanding comprehensive performance including accuracy, robustness, low workload, and better anti-multipath fading.

## Figures and Tables

**Table 1:** Performance comparison between different algorithms and input data.

Algorithm	KNN		WKNN		RFFP
InPut Data	RSSI	CSI	RSSI	CSI	CSI
MAE in chamber (m)	1.7085	0.7608	1.1255	0.1767	0.1033
MAE in office, LOS (m)	2.8724	1.8421	2.1812	0.8164	0.1708
MAE in office, NLOS (m)	2.9463	1.7782	2.3071	1.0517	0.4033

**Table 2:** Performance comparison between different wireless channel circumstances.

Algorithm	J48	CART	REPTree	Ensemble of BPNNs	RFFP
Accuracy (chamber)	91.64%	92.08%	89.68%	96.36%	96.04%
Accuracy (office, LOS)	71.44%	67.68%	63.76%	92.52%	93.12%
Accuracy (office, NLOS)	65.74%	60.20%	61.13%	77.88%	84.18%

**Table 3:** Performance comparison between different numbers of access points (APs).

Number of APs	Accuracy	OOBE	MAE (m)	TP Rate	FP Rate	Precision	F-Measure	Build Time (s)
1	75.28%	13.44%	0.5864	0.753	0.01	0.758	0.751	27.42
2	89.92%	9.76%	0.2548	0.899	0.004	0.902	0.899	28.63
3	92.48%	7.2%	0.1930	0.925	0.03	0.927	0.925	29.5
4	93.12%	6.56%	0.1708	0.931	0.03	0.934	0.931	30.3
